# Mapping Patterns and Trends in the Spatial Availability of Alcohol Using Low-Level Geographic Data: A Case Study in England 2003–2013

**DOI:** 10.3390/ijerph14040406

**Published:** 2017-04-12

**Authors:** Colin Angus, John Holmes, Ravi Maheswaran, Mark A. Green, Petra Meier, Alan Brennan

**Affiliations:** 1 School of Health and Related Research, Regent Court, University of Sheffield, Sheffield S1 4DA, UK; john.holmes@sheffield.ac.uk (J.H.); r.maheswaran@sheffield.ac.uk (R.M.); mark.green@liverpool.ac.uk (M.A.G.); p.meier@sheffield.ac.uk (P.M.); a.brennan@sheffield.ac.uk (A.B.); 2 School of Environmental Sciences, Jane Herdman Building, University of Liverpool, Liverpool L69 3GP, UK

**Keywords:** alcohol, availability, socioeconomic status, licensing, public health policy, health inequalities

## Abstract

Much literature examines the relationship between the spatial availability of alcohol and alcohol-related harm. This study aims to address an important gap in this evidence by using detailed outlet data to examine recent temporal trends in the sociodemographic distribution of spatial availability for different types of alcohol outlet in England. Descriptive analysis of measures of alcohol outlet density and proximity using extremely high resolution market research data stratified by outlet type and quintiles of area-level deprivation from 2003, 2007, 2010 and 2013 was undertaken and hierarchical linear growth models fitted to explore the significance of socioeconomic differences. We find that overall availability of alcohol changed very little from 2003 to 2013 (density +1.6%), but this conceals conflicting trends by outlet type and area-level deprivation. Mean on-trade density has decreased substantially (−2.2 outlets within 1 km (Inter-Quartile Range (IQR) −3–0), although access to restaurants has increased (+1.0 outlets (IQR 0–1)), while off-trade access has risen substantially (+2.4 outlets (IQR 0–3)). Availability is highest in the most deprived areas (*p* < 0.0001) although these areas have also seen the greatest falls in on-trade outlet availability (*p* < 0.0001). This study underlines the importance of using detailed, low-level geographic data to understand patterns and trends in the spatial availability of alcohol. There are significant variations in these trends by outlet type and deprivation level which may have important implications for health inequalities and public health policy.

## 1. Introduction

Alcohol consumption is associated with a wide range of health and societal harms [1,2] and reducing the spatial availability of alcohol has been proposed as a key policy approach to reduce these harms [3]. Alcohol availability includes spatial, temporal, economic and psychological aspects and particular focus has been given to spatial availability, given that many jurisdictions limit retailers’ ability to sell alcohol through licensing or monopoly systems [4]. Proposals to restrict spatial availability arise from a large body of scientific literature exploring the relationship between spatial availability, alcohol consumption and harm. Recent reviews of this literature provide evidence of a positive association between spatial availability and outcomes including alcohol consumption, alcohol-related mortality, drink-driving, assault, domestic abuse and public nuisance [5,6,7,8].

There has been little investigation, however, of how alcohol availability, as well as the types of outlets that comprise that availability, have varied over time by social characteristics. Previous studies in the USA, Australia and Scotland have looked separately at the relationship between alcohol outlets and measures of area-level socioeconomic deprivation (e.g., [9,10,11,12]) or temporal trends in overall availability (e.g., [13,14]), but not how they interact. Such studies also only infrequently explore how temporal trends and social patterning of availability may vary between different categories of outlet and, particularly, more detailed categories than on-trade vs. off-trade. A more nuanced understanding may be important in explaining patterns of alcohol-related harm as different outlet types can hold greater appeal for different population groups [15] and have different potential harm consequences [16]. For example, nightclub availability has little relevance to contemporary health harm in older age groups while outlet categories with problematic sales practices may plausibly be particularly concentrated in less organised communities [17]. Improving our understanding of these trends in alcohol availability is necessary to inform effective policy design, particularly in understanding the targeting of different policies on different population groups and areas.

A deeper understanding of the social patterns of availability is also important in the context of the global focus on reducing health inequalities [18]. Alcohol is a key contributor to these inequalities [19,20], with significantly higher rates of alcohol-related harm in more deprived areas [21]. Several studies have found that availability is also higher in areas of deprivation [10,22,23], although this relationship may not hold for all types of outlet or in areas of greatest deprivation [9,12]. Identifying temporal trends in the sociodemographic patterning of availability may help to better explain trends in alcohol-related health inequalities as well as informing the design of future interventions to address these inequalities.

A further feature of the alcohol availability literature to date is that the vast majority of studies have used measures derived from outlet data with relatively modest geographic precision such as U.S. zip codes or block groups, local government regions or census tracts whose populations may number several thousand inhabitants [8]. Data with greater spatial resolution in combination with multiple time points and a more disaggregated outlet classification would enable a detailed exploration of the interrelationship between spatial availability, socioeconomic deprivation, different outlet types and temporal trends. This study harnesses the availability of such detailed data for England for the period from 2003 to 2013 to quantify these links and place them in the context of alcohol policy over this period. In doing so, it aims to provide a detailed understanding of the changing face of alcohol availability in England between 2003 and 2013 and its potential impact on existing inequalities in alcohol-related harms.

## 2. Materials and Methods

### 2.1. Data

The UK Government does not hold a central database of licensed premises; these are held in inconsistent formats by the 326 local licensing boards in England and are frequently not readily accessible to the public. We therefore obtained data on the location and outlet type of all premises selling alcohol in England for the years 2003, 2007, 2010 and 2013 from market research companies CGA Strategy (CGA) and Nielsen. On-trade (where alcohol is sold for consumption on the premises, e.g., pubs and restaurants) outlet data was provided by CGA, with Nielsen providing data for the off-trade (where alcohol is sold for consumption off the premises, e.g., supermarkets). Both datasets include the full postcode for each outlet—UK postcodes vary in size but typically contain around 15 residential and/or commercial addresses and have a mean area of 0.14 km^2^ (0.054 miles^2^) [24].

Nielsen and CGA obtain their data from a wide range of sources including local licensing boards, third party business directories and the Royal Mail Postal Address File, as well as directly from alcohol suppliers and retailers. The databases are updated monthly and the data used here are for January in each year, except for 2013 data which is for December. Premises are contacted by telephone to confirm that they continue to trade and sell alcohol and in each year, it is estimated that 98% of all outlets are within the dataset with around 85% of all included outlets having been actively confirmed to be trading [25]. This data provides a clearer and more current picture of spatial availability then data from local licensing boards, which may be infrequently updated and include outlets which are no longer trading as well as being prohibitively difficult to obtain for the entire country and potentially of low quality [10,13].

### 2.2. Outlet Measures

The CGA on-trade data contains a 69-category outlet classification, with categories such as “Community/wet led/local pub”, “Casino”, “Hotel” and “Indian restaurant”. There have been minor revisions to the 69 categories between 2003 and 2013 and therefore in order to maintain consistency across all time points and in line with categorisations used in existing international studies, the on-trade classification was collapsed into three categories for analysis:

- Pubs, bars and nightclubs (café bars, wine bars and both wet- and dry-led pubs which focus on alcohol and food respectively)
- Restaurants
- All other on-trade outlets (including sports and social clubs, hotels, casinos and conference venues)

The off-trade data supplied by Nielsen included the full postcode for each outlet as well as an eight-category outlet classification, including categories such as “Convenience store”, “Specialist off-licence” and “Wholesaler”. This classification has been revised significantly between 2010 and 2013, with a number of outlets changing category due to improved data collection processes, including the supply of data directly by a number of major retailers. Furthermore, two new outlet categories have been added: “Wholesaler” and “Forecourt”, which were previously classified as “Other”. In order to address these revisions, outlets present in the 2013 data were reclassified as the 2013 outlet type in all previous years in which they were present. Thus an outlet appearing in 2013 data as a “Supermarket” and as a “Convenience store” in previous years would be classified as a “Supermarket” across all years. The only exceptions to this remapping were outlets classified prior to 2013 as “Independent”. Between 2010 and 2013 there were significant changes to this sector of the alcohol market, with a number of major national chains, notably “Thresher” and “Victoria Wines”, ceasing to trade and many of their sites being redeveloped as convenience stores. It was therefore assumed that any change in categorisation of these outlets represented a genuine change in use of the site. These revisions to the data were agreed with CGA/Nielsen in advance of any analyses being performed. After revisions, all off-trade outlets were grouped into three categories for analysis:

- Supermarkets (large grocery store providing many services, usually over 280 m^2^ in floor area)
- Convenience store (smaller grocery store providing limited/essential food and drink, usually under 280 m^2^ in area—note that in the UK the vast majority of grocery stores sell alcohol)
- All other off-trade outlets (including specialist off-licenses, corner shops and petrol station forecourts)

### 2.3. Availability Measures

We obtained geospatial data from the Office for National Statistics [26] giving the detailed grid reference (maximum resolution 1 m) of the address-weighted centroid of each postcode and this data was used to allocate a physical location to each of the more than 150,000 outlets in each of the four years. Two measures of availability were then constructed for each of the 1.3 million residential postcodes in England (as defined in the 2011 census), which form the units of analysis for this study.

- *Outlet proximity*; the Euclidean (straight line) distance from the centroid of a postcode to the nearest centroid of a postcode containing an outlet of a given type.
- *Outlet density*; the total number of outlets of a given type in a postcode whose centroid lay within a 1 km radius of the postcode centroid.

### 2.4. Area-Level Measure

The lowest level of geography in England and Wales at which data on area-level deprivation is available is the Lower Super Output Area (LSOA). Each LSOA typically consists of between 400 and 1200 households with a combined population of 1000–3000 (2011 Census) [24]. In all there were 32,844 LSOAs in England at the last boundary revision in 2011. LSOA-level deprivation was measured using the Index of Multiple Deprivation (IMD) [27], the preferred measure used by the UK government. This is a composite measure which is updated approximately every three years (2004, 2007, 2010 and 2015), which scores and ranks every LSOA based on seven domains including income, employment and health. Each year of outlet data was matched to the closest published year of IMD data and deprivation quintiles were calculated and assigned to each postcode based on the overall IMD ranking of the LSOA within which it sits.

### 2.5. Statistical Analyses

All data was processed and analysed using R and Stata statistical software [28,29]. The significance of temporal trends in outlet density was analysed using linear growth curve models fitted with the xtmixed function in Stata. LSOA-level density measures were log-transformed and associated with quintiles of deprivation. Variation between LSOAs at baseline and individual LSOA time trends were modelled as hierarchical random intercept-random slope mixed-effects models with nested random effects at region and postcode level. Separate models were fitted by outlet type, treating IMD quintile as a categorical variable so as not to impose a linear gradient on model results.

## 3. Results

Table 1 presents the total number of outlets selling alcohol, by category, in each of the four years 2003, 2007, 2010 and 2013. There were a total of 153,024 outlets in England in 2013, equating to 3.6 outlets for every 1000 adults. The single most common outlet type in 2013 was pubs, bars and nightclubs, accounting for a third (33%) of all outlets. Restaurants accounted for 14% of the total, with 24% of outlets being other on-trade types such hotels and sports and social clubs. The remaining 30% were off-trade outlets of which the majority were convenience stores (38% of off-trade, 11% of all outlets) or other off trade outlets (51% of off-trade, 15% of all outlets). Supermarkets represented a small proportion of the overall number of outlets (4%).

Between 2003 and 2013, the total number of outlets fell by 2% (−2610 outlets), but this reflects a 12% fall (−14,678) in the number of on-trade outlets being offset by a 36% (+12,068) increase in the number of off-trade outlets. Within on-trade outlets, restaurant numbers have increased by 16% (+3023) while all other outlet types have declined in number. All off-trade outlet types have increased in number, with the largest increases being among convenience stores, whose numbers have more than doubled (+8384; 104%). These changes have not occurred uniformly across the period of analysis for all outlet types, for example supermarket numbers increased most between 2007 and 2010, while the rise in convenience stores is concentrated between 2010 and 2013.

**Figure 1:**
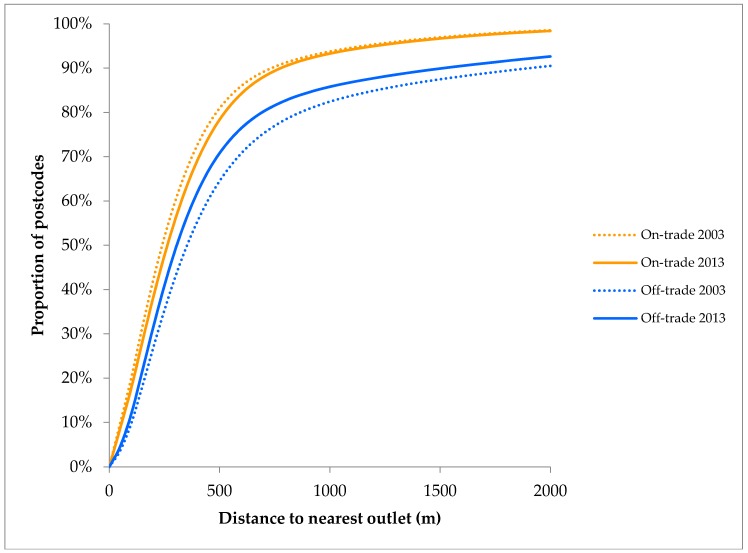
Distribution of distances to nearest outlet (2013).

Table 2 presents the four calculated availability measures, by outlet type, for each year of data. These results show that in 2013 the mean distance to an on-trade outlet was 383 m (Inter-Quartile Range (IQR) 135–458), with the equivalent figure for off-trade outlets being 610 m (IQR 167–569). Figure 1 shows these distributions visually for 2003 and 2013, illustrating that 94% of English postcodes were within 1 km and 85% within 500 m of an outlet selling alcohol.

The results for outlet density show that postcodes had an average of 31 outlets within 1 km of their centroid in 2013 (IQR 6–33), including 10 pubs (IQR 2–9), 6 restaurants (IQR 0–4) and 9 outlets selling alcohol for consumption off the premises (IQR 2–12). Across all measures, particularly density in the on-trade, there is considerable variation across the country.

Over the period from 2003 to 2013, the average distance to an outlet selling alcohol has increased by just 5 m, although this masks very conflicting patterns within outlet categories. As illustrated by Figure 1, distances to the nearest on-trade outlet have increased, while decreasing for off-trade outlets. Breaking this down further, the average distance to the nearest pub has increased (from 471 m to 501 m), whilst for restaurants, supermarkets, convenience stores and other off-trade outlet types it has decreased (e.g., convenience stores from 1.4 km to 1.0 km). Similar patterns are observed in outlet density, with an average of 0.8 fewer pubs (−7.9%), 1 additional restaurant (+19.2%) and 1.8 additional convenience stores (+118.4%) within 1 km.

**Figure 2:**
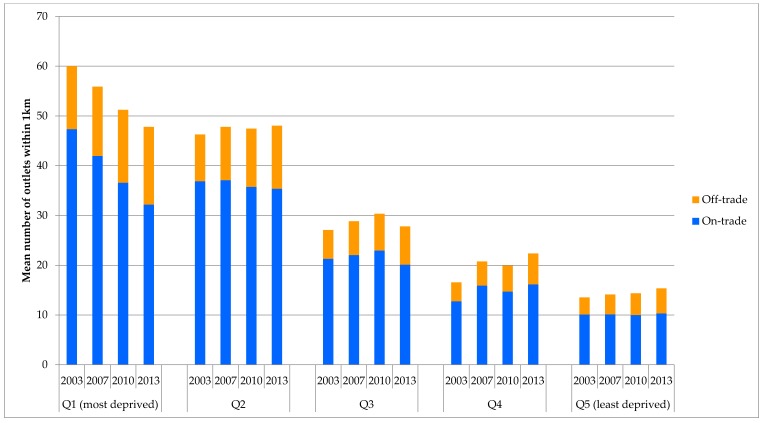
Socioeconomic gradients in mean outlet density over time.

Table 3 presents the results for outlet density stratified by quintiles of deprivation. There is a clear social gradient in outlet density with higher densities of outlets, particularly in the on-trade, in more deprived postcodes. Analysis of the temporal patterns in this gradient shows significant variation in trends for different sections of the socioeconomic spectrum. On-trade availability has reduced substantially (−32.0%) in the most deprived quintile, compared to the second most deprived quintile (−4.1%), with the highest levels of on-trade availability shifting to this second most deprived quintile by 2013. Over the same period outlet density increased in the second least deprived quintile (+26.8%). Off-trade availability has increased across all quintiles, but with the greatest absolute increase in the second most deprived quintile (+3.3 outlets). These trends are illustrated in Figure 2 which also highlights that overall availability has reduced since 2003 in the most deprived quintile while increasing everywhere else and that by 2013 these countervailing trends had led to the disappearance of any difference in mean overall outlet density between the two most deprived quintiles.

**Figure 3:**
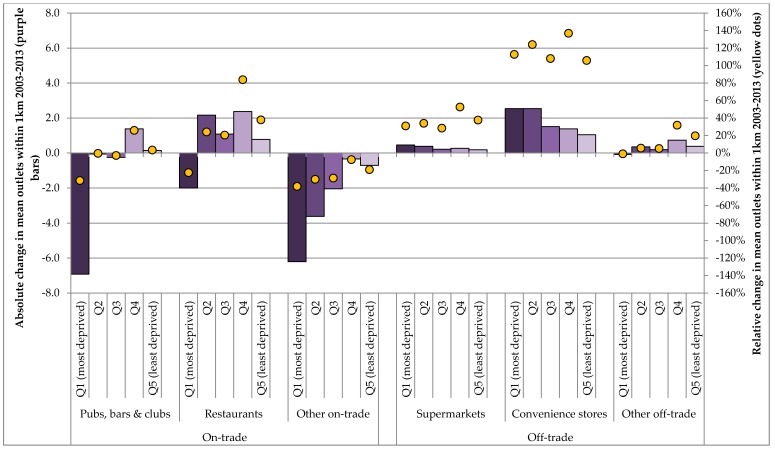
Absolute (bars) and relative (dots) changes in outlet density by deprivation and outlet category 2003–2013.

These temporal trends are further unpacked in Figure 3 and Table 4, which illustrate absolute and relative changes in outlet density across the period of analysis by quintiles of deprivation and outlet type. These highlight that the most deprived quintile of postcodes have seen large absolute and relative reductions in the density of all on-trade outlet types while the second least deprived quintile has seen the largest increase, or smallest decreases. The most striking deprivation gradient is in changes to access to pubs, bars and clubs, which has fallen substantially in the most deprived quintile, increased in the second-least deprived and barely changed in other areas. Access to restaurants has also fallen in the most deprived quintile, while increasing in all other areas. In contrast, increases in off-trade outlets are more uniform, although the two most deprived quintiles have seen the largest absolute increases in both supermarket and convenience store access, while the second-least deprived quintile has seen the largest relative increase across all off-trade outlet types.

It is interesting to note (underlying data in Supplementary Materials Table S1) that if the deprivation quintile of each postcode is fixed at its baseline (2003 level) the conflicting trends in the two most deprived quintiles disappear. This illustrates that these differing trends are being driven by postcodes moving between quintiles of deprivation rather than changes in availability in postcodes which have remained within the same quintile. 15.2% of postcodes in the most deprived quintile in 2003 had moved to the second quintile by 2013. These postcodes had significantly higher baseline availability than those that remained in the most deprived quintile (mean overall density 92.6 vs. 53.4, Mann-Whitney U-test *p* < 0.0001) and saw an increase in availability between 2003 and 2013 while overall availability in postcodes remaining in the lowest quintile reduced (mean change in overall density +6.9 vs. −3.6 Mann-Whitney U-test *p* < 0.0001).

Results of the fitted growth models relating deprivation to outlet type-specific density can be found in Supplementary Materials Table S2. These are separated into the modelled effects on the intercept (i.e., the log of outlet density at baseline in 2003) and on the slope (i.e., the change in the log of outlet density with each additional year subsequent to 2003). Results are consistent with those already presented, showing greater baseline availability across all outlet types in the most deprived areas. Temporal trends are strongest in the most deprived areas for all outlet types except for restaurants where the greatest changes are in the least deprived areas. 

For all outlet types, deprivation is a highly significant (*p* < 0.0001) predictor of both baseline outlet density and of changes in density over time. For example, moving from the most to the second most-deprived quintile is associated with a reduction of 25.0% (i.e., $100\times (e^{-0.288}-1)$) in the density of convenience stores in 2003 and a 1.26% reduction in the change in outlet density per year subsequently. In addition to being jointly significant, all deprivation coefficients for both the effect on the intercept and the slope are individually significant across all outlet types, with the exception of the slope coefficients for other off-trade outlets and restaurants, for which the second most deprived quintile is not significantly different from the most deprived quintile.

## 4. Discussion

Our study uses detailed, longitudinal, data with high geographic precision to quantify the availability of alcohol in England; how this varies by deprivation and over time and whether these patterns are different for different types of outlet. Results show that alcohol is available within very short travelling distances for the vast majority of the population and that this has hardly changed over time (+5 m). In contrast, the nature of alcohol availability has altered with a notable shift from on-trade to off-trade. Availability of alcohol from pubs, bars and nightclubs, the single largest category of outlet, has decreased by 8% between 2003 and 2013, while the availability of off-trade alcohol has increased substantially, with convenience store numbers more than doubling over this period. Breaking these results down by deprivation quintiles shows more marked differences across the socioeconomic spectrum, with a clear social gradient in availability whereby the most deprived areas have the greatest exposure to alcohol outlets. This gradient may be explained to some extent by the fact that more deprived areas are more likely to be more urban and have higher population density, although stratifying results by urban/rural status does not alter our conclusions. Furthermore, whilst greater availability in deprived areas may be driven by higher population density and greater provision of retail outlets in general [30], this does not alter the fact that inhabitants of these areas are exposed to very high levels of alcohol availability The observed socioeconomic patterns have shifted somewhat over time, with overall availability decreasing sharply in the most deprived quintile, driven by falling on-trade outlet numbers. These trends are very different in the second most deprived quintile which has seen overall availability rise, primarily through increasing access to restaurants and off-trade outlets.

In addition to highlighting the value of using such detailed data, our study also provides the first detailed examination of spatial alcohol availability in England, over a period when there have been significant political and cultural shifts. Most notably, significant deregulatory changes to the English licensing system were introduced in 2005 via the Licensing Act (2003) [31]. This law made it substantially easier to apply for new off-trade than new on-trade licenses [32]. The broader impact of the act has been widely discussed (e.g., [33,34,35,36]), but the impact of these changes on the availability of alcohol have not previously been quantified at a national level. More recently, legislative changes have placed an increased responsibility for public health on Local Authorities and regulation of alcohol retail licenses is a key mechanism by which they can exercise this responsibility. This level of local control is common internationally and an understanding of the current landscape of availability is fundamental to consideration of how best to harness these available powers to improve public health. Over the same period, patterns of living have changed, with changing working hours and work intensity [37] and the retail market has changed with it, with a huge rise in smaller so-called “metro supermarkets” dominated by a few major retailers [38]. The analysis presented here illustrates the implications these changes have had for the spatial availability of alcohol.

Our study represents one of the most detailed analyses yet undertaken of outlet availability and deprivation, disaggregating different outlet types and considering a range of measures of availability; however there are a number of limitations. Firstly, whilst the low geographic level of the outlet location data allows more fine-grained analysis than much previous work in this area, measures of availability tied to a single location cannot fully represent the availability experienced by individuals living within each postcode in the course of their everyday lives (e.g., the supermarket passed every day on one’s commute to work). In addition, our measures of availability were calculated using Euclidian distances between postcode centroids, which may not fully capture barriers to movement (e.g., rivers and road networks [39]) and do not account for the differing size of outlets. There are also limitations in the data itself, for example there may be errors in the outlet classifications provided by the data owners, although this should be balanced against the fact that similarly detailed data is not available from alternative sources. Secondly, area-level measures such as deprivation are not typically available at very low levels of geography and we have therefore had to assume homogeneity in the deprivation level of all postcodes within each LSOA, although social homogeneity is a factor considered in the determination of LSOA boundaries [40]. There is also a slight temporal misalignment for the oldest and newest years of data, with data from 2003 and 2013 matched to deprivation levels calculated in 2004 and 2015 respectively. Finally our data does not include outlet opening hours and we have therefore only examined spatial, rather than temporal or other aspects of availability. This may be significant in the context of the Licensing Act (2003) which introduced late night and extended licenses, leading to greater temporal availability across the country, although there is some evidence to suggest that the impact of this legislation on public health and disorder may have been to temporally displace rather than increase alcohol-related harm [41,42].

The analysis presented here focuses on England; however there are important implications for research which apply beyond the geographical scope of this study. Not only does this study illustrate the value of detailed, longitudinal, data in characterising alcohol availability, it also highlights the extent to which analyses which look only at highly aggregated outlet classifications may miss important changes in the underlying structure of the availability landscape. Further research may seek to explore these changes in greater detail, using even more disaggregated classifications, particularly where outlet types can be identified which may be particularly problematic from a public health perspective, such as those selling particularly cheap alcohol, or failing to adhere to or enforce sales restrictions. Similarly, given the strong links between area-level deprivation and health, an understanding of the relationship between deprivation and availability is critical public health intelligence and valuable for design, targeting and evaluations of interventions aiming to address the impact of changes in availability on health. A failure to fully capture some aspects of this complexity may partially explain some of the apparently conflicting findings in recent studies linking availability to harm outcomes [43,44]. For example, different subgroups of the population are likely to be affected in different ways by different forms of alcohol availability and the development of more specific, disaggregated measures of availability may help to better understand the complexity of the relationships between availability, alcohol consumption and harm. We also only explore geographical accessibility as our measure of alcohol availability and therefore it will be important for future research to also consider associated issues such as: differences in opening and closing hours, pricing, availability of high strength alcohol, and marketing. These will each affect the type of alcohol accessibility within a local neighbourhood and may produce different alcohol-related harms or vary by level of deprivation, particularly when considered alongside variations in the characteristics of the local population, such as their sociodemographic composition, access to transport and the availability of healthcare services.

There are also important insights from a public health perspective in the analysis of detailed data such as this. Our findings show that overall the spatial availability of alcohol has decreased significantly in the most deprived areas, which are the areas which suffer the highest rates of alcohol-related harm [19]. This positive trend, however, may be counteracted to some extent by the fact that off-trade availability has increased across all areas and the price of alcohol in the off-trade is generally considerably lower [45], meaning that cheap alcohol is now more readily available in England than at any time in the past decade. The shifting of greatest spatial availability from the most towards the second-most deprived quintile, something that echoes findings elsewhere [12], is also interesting in the context that this appears to be driven by high and increasing availability in areas which have moved from the lowest quintile while availability has decreased in areas which are moving in the other direction. That is to say that new outlets may be opening in areas which are upwardly mobile, while outlets are closing more quickly in areas in which deprivation is increasing—suggesting, at least in part, that changes in availability may be demand-led. Future research may wish to develop this hypothesis further, or to examine alternative explanations and factors driving the changes in availability described here.

## 5. Conclusions

This study presents an illustration of the value of detailed, longitudinal, data on the location and nature of alcohol outlets in enabling researchers and public health policy makers to unpack patterns and trends in the availability of alcohol, which may be highly divergent across outlet types and areas. Failure to account for heterogeneity in outlet types or socioeconomic patterns in availability may lead to misleading research findings and the adoption of suboptimal or even damaging public health policies. 

## Figures and Tables

**Table 1:** Outlet counts by category and year.

Outlet Category		2003	2007	2010	2013	Change 2003–2013	% Change 2003–2013
**Population in millions (aged 18+)**		38.8	40.2	41.4	42.4	3.5	9%
**On-trade**	**Pubs, bars and nightclubs**	55,105	56,204	53,487	49,940	−5165	−9%
	**Restaurants**	18,410	18,849	19,160	21,433	3023	16%
	**Other on-trade**	48,727	45,848	43,115	36,191	−12,536	−26%
**Off-trade**	**Supermarkets**	4417	5101	6072	5859	1442	33%
	**Convenience stores**	8083	11,225	11,901	16,467	8384	104%
	**Other off-trade**	20,892	22,166	22,874	23,134	2242	11%
**On-trade**		122,242	120,901	115,762	107,564	−14,678	−12%
**Off-trade**		33,392	38,492	40,847	45,460	12,068	36%
**All outlets**		155,634	159,393	156,609	153,024	−2610	−2%

**Table 2:** Average spatial alcohol availability measures across all English postcodes by outlet category and year.

Outlet Category		Mean Availability (Inter-Quartile Range)				Change from 2003 to 2013 (IQR)	Mean % Change from 2003 to 2013
		2003	2007	2010	2013		
**Outlet proximity (distance to nearest outlet (m))**	**Pubs, bars & nightclubs**	471 (167–566)	467 (167–565)	484 (174–590)	501 (182–613)	31 (0–0)	6.6%
	**Restaurants**	1469 (429–1843)	1477 (423–1831)	1489 (425–1822)	1424 (407–1712)	−45 (−104–11)	−3.1%
	**Other on-trade**	653 (206–717)	667 (214–731)	679 (222–747)	724 (241–797)	71 (0–51)	10.9%
	**Supermarkets**	2146 (626–2485)	1958 (575–2199)	1888 (535–2086)	1904 (549–2133)	−242 (0–0)	−11.3%
	**Convenience stores**	1397 (385–1492)	1148 (324–1183)	1122 (314–1139)	997 (272–953)	−400 (−349–0)	−28.6%
	**Other off-trade**	1039 (234–1040)	955 (226–949)	946 (223–931)	891 (225–929)	−149 (0–0)	−14.3%
	**All on-trade**	357 (121–423)	358 (123–426)	368 (128–439)	383 (135–458)	26 (0–0)	7.3%
	**All off-trade**	749 (189–692)	668 (177–617)	657 (174–603)	610 (167–569)	−139 (−5–0)	−18.6%
	**All outlets**	318 (102–362)	315 (102–356)	319 (104–360)	323 (106–363)	5 (0–0)	1.6%
**Outlet density (no. of outlets within 1 km)**	**Pubs, bars & nightclubs**	10.7 (2–10)	11 (2–11)	10.2 (2–10)	9.8 (2–9)	−0.8 (−1–0)	−7.9%
	**Restaurants**	5.3 (0–4)	5.7 (0–4)	5.8 (0–4)	6.4 (0–4)	1 (0–1)	19.2%
	**Other on-trade**	8.3 (1–9)	7.6 (1–8)	7.1 (1–8)	6 (1–6)	−2.4 (−3–0)	−28.3%
	**Supermarkets**	0.8 (0–1)	1 (0–2)	1.2 (0–2)	1.1 (0–2)	0.3 (0–0)	35.6%
	**Convenience stores**	1.5 (0–2)	2.1 (0–3)	2.3 (0–3)	3.3 (1–4)	1.8 (0–2)	118.4%
	**Other off-trade**	4.4 (0–6)	4.6 (1–6)	4.8 (1–6)	4.7 (1–6)	0.4 (0–1)	8.2%
	**All on-trade**	24.3 (4–24)	24.3 (4–23)	23.1 (3–22)	22.2 (3–20)	−2.2 (−3–0)	−8.9%
	**All off-trade**	6.7 (1–9)	7.7 (2–10)	8.3 (2–11)	9.1 (2–12)	2.4 (0–3)	36.1%
	**All outlets**	31 (5–33)	32 (6–34)	31.3 (6–33)	31.3 (6–33)	0.2 (−2–2)	0.8%

**Table 3:** Average outlet density (number of outlets within 1 km of postcode centroid) by deprivation and year.

Outlet Type by Deprivation Quintile		Mean Outlet Density (IQR)				Mean Change from 2003 to 2013	% Change in Mean from 2003 to 2013
		2003	2007	2010	2013		
**On-trade**	**Q1 (most deprived)**	47.3 (10–52)	42 (10–47)	36.6 (9–39)	32.2 (7–34)	−15.1	−32.0%
	**Q2**	36.9 (7–36)	37.1 (7–34)	35.8 (7–32)	35.4 (5–29)	−1.5	−4.1%
	**Q3**	21.3 (3–22)	22.1 (3–21)	23 (2–20)	20.1 (2–17)	−1.2	−5.6%
	**Q4**	12.7 (2–14)	15.9 (2–14)	14.7 (2–13)	16.2 (2–14)	3.4	26.8%
	**Q5 (least deprived)**	10.1 (3–12)	10.1 (3–12)	10 (3–11)	10.3 (3–12)	0.2	2.2%
**Off-trade**	**Q1 (most deprived)**	12.7 (6–18)	13.9 (7–19)	14.6 (7–20)	15.6 (8–21)	2.9	23.1%
	**Q2**	9.4 (3–13)	10.7 (4–14)	11.7 (4–16)	12.7 (4–17)	3.3	34.9%
	**Q3**	5.8 (1–8)	6.8 (1–9)	7.4 (1–10)	7.7 (1–11)	1.9	33.2%
	**Q4**	3.8 (0–5)	4.9 (0–7)	5.2 (0–7)	6.2 (1–8)	2.4	62.4%
	**Q5 (least deprived)**	3.4 (1–5)	4 (1–6)	4.4 (1–6)	5 (2–7)	1.6	47.2%
**All outlets**	**Q1 (most deprived)**	60 (17–70)	55.9 (17–67)	51.2 (17–60)	47.8 (16–55)	−12.2	−20.4%
	**Q2**	46.3 (12–49)	47.8 (12–48)	47.4 (12–48)	48.1 (11–47)	1.8	3.8%
	**Q3**	27.1 (4–30)	28.9 (4–31)	30.4 (4–30)	27.8 (3–29)	0.7	2.6%
	**Q4**	16.6 (2–19)	20.8 (3–21)	19.9 (2–21)	22.4 (3–23)	5.8	35.0%
	**Q5 (least deprived)**	13.5 (4–17)	14.1 (4–17)	14.4 (4–17)	15.4 (5–18)	1.8	13.6%

**Table 4:** Outlet density by deprivation quintile and outlet type.

Outlet Category by Deprivation Quintile			Mean Outlet Density				Change from 2003 to 2013	% Change from 2003 to 2013
			2003	2007	2010	2013		
**On-trade**	**Pubs, bars & clubs**	**Q1 (most deprived)**	14.9	13.7	11.8	10.4	−4.5	−30.2%
		**Q2**	10.9	11.0	10.6	10.6	−0.3	−2.9%
		**Q3**	6.4	6.8	6.7	6.2	−0.2	−2.7%
		**Q4**	4.1	4.8	4.6	5.1	1.0	23.1%
		**Q5 (least deprived)**	3.5	3.6	3.5	3.7	0.1	4.0%
	**Restaurants**	**Q1 (most deprived)**	9.0	7.5	6.8	7.0	−2.0	−22.3%
		**Q2**	8.9	9.5	9.8	11.1	2.2	24.2%
		**Q3**	5.3	5.7	6.8	6.4	1.1	20.6%
		**Q4**	2.8	4.4	4.2	5.2	2.4	83.9%
		**Q5 (least deprived)**	2.0	2.1	2.3	2.8	0.8	38.0%
	**Other on-trade**	**Q1 (most deprived)**	16.3	14.0	12.6	10.1	−6.2	−38.1%
		**Q2**	12.1	10.9	10.1	8.4	−3.6	−30.0%
		**Q3**	7.1	6.7	6.5	5.1	−2.0	−28.6%
		**Q4**	4.6	4.9	4.4	4.2	−0.3	−7.5%
		**Q5 (least deprived)**	3.7	3.6	3.4	3.0	−0.7	−18.9%
**Off-trade**	**Supermarkets**	**Q1 (most deprived)**	1.5	1.7	2.0	1.9	0.5	31.1%
		**Q2**	1.1	1.3	1.6	1.5	0.4	34.0%
		**Q3**	0.8	0.9	1.1	1.0	0.2	28.6%
		**Q4**	0.5	0.6	0.7	0.8	0.3	52.7%
		**Q5 (least deprived)**	0.5	0.6	0.7	0.7	0.2	37.8%
	**Convenience stores**	**Q1 (most deprived)**	2.2	3.2	3.3	4.8	2.5	112.8%
		**Q2**	2.0	3.0	3.2	4.6	2.5	124.1%
		**Q3**	1.4	2.0	2.1	2.9	1.5	108.1%
		**Q4**	1.0	1.5	1.6	2.4	1.4	137.0%
		**Q5 (least deprived)**	1.0	1.3	1.4	2.0	1.0	105.9%
	**Other off-trade**	**Q1 (most deprived)**	8.9	9.1	9.3	8.9	−0.1	−0.9%
		**Q2**	6.2	6.4	6.8	6.6	0.4	5.7%
		**Q3**	3.6	3.9	4.2	3.8	0.2	5.3%
		**Q4**	2.3	2.7	2.9	3.0	0.7	31.9%
		**Q5 (least deprived)**	1.9	2.1	2.3	2.3	0.4	19.8%

## Supplementary Materials

The following are available online at www.mdpi.com/1660-4601/14/4/406/s1, Table S1: Mean outlet density by deprivation and year with postcode deprivation fixed at baseline (2003) level, Table S2: Hierarchical growth model results: effects of deprivation on logged outlet density.
