# Supplementary material for: Mapping Patterns and Trends in the Spatial Availability of Alcohol Using Low-Level Geographic Data: A Case Study in England 2003–2013
Source: Int J Environ Res Public Health. 2017 Apr 12;14(4):406. doi: 10.3390/ijerph14040406 (PMC5409607; doi:10.3390/ijerph14040406)
Supplement: Supplementary file 1 [file ijerph-14-00406-s001.pdf]

Table S1. Mean outlet density by deprivation and year with postcode deprivation fixed at baseline (2003) level.

Outlet Type by Deprivation Quintile		Mean Outlet Density (IQR) 2003	2007	2010	2013	Change from 2003–2013 (IQR)	Mean % Change from 2003–2013
On-trade	**Q1 (most deprived)**	40.2 (10–44)	38.9 (9–42)	36.6 (9–37)	34.6 (7–34)	−5.6 (−9–−1)	-14.0%
	**Q2**	31.9 (7–31)	31.7 (7–30)	30.4 (6–28)	29.5 (6–26)	−2.4 (−5–0)	-7.6%
	**Q3**	18.8 (3–20)	18.8 (3–19)	18.3 (3–18)	17.8 (2–17)	−1 (−3–0)	-5.4%
	**Q4**	11.5 (2–13)	11.4 (2–13)	11 (2–12)	10.7 (2–11)	−0.8 (−2–0)	-7.2%
	**Q5 (least deprived)**	9.3 (3–11)	9.3 (3–11)	9 (3–11)	8.8 (2–10)	−0.5 (−1–0)	-5.4%
Off-trade	**Q1 (most deprived)**	12.7 (6–18)	14.3 (7–19)	15.4 (7–21)	17.1 (8–23)	4.4 (1–6)	34.8%
	**Q2**	9.4 (3–13)	10.9 (4–15)	11.8 (5–16)	13 (5–17)	3.6 (1–5)	37.9%
	**Q3**	5.8 (1–8)	6.7 (1–9)	7.2 (1–10)	7.9 (1–11)	2.1 (0–3)	36.3%
	**Q4**	3.8 (0–5)	4.5 (0–7)	4.8 (0–7)	5.2 (0–8)	1.4 (0–2)	36.6%
	**Q5 (least deprived)**	3.4 (1–5)	4 (1–6)	4.3 (1–6)	4.6 (1–7)	1.2 (0–2)	35.1%

Table S2. Hierarchical growth model results: effects of deprivation on logged outlet density.

Model Covariate and Level		Pubs, Bars & Nightclubs	Restaurants	Other on-Trade	Supermarkets	Convenience Stores	Other off-Trade
		Model Estimates (Standard Errors)					
Baseline (2003) logged outlet density (intercept)		1.225 (0.215)	−2.213 (0.503)	0.824 (0.236)	-3.135 (0.182)	−1.584 (0.266)	−0.354 (0.34)
		p < 0.0001	*p* < 0.0001	*p* < 0.0001	*p* < 0.0001	*p* < 0.0001	*p* = 0.298
Effect on intercept	IMD Q1 (most deprived)	ref.	ref.	ref.	ref.	ref.	ref.
	IMD Q2	−0.201 (0.003)	−0.239 (0.006)	−0.284 (0.004)	−0.256 (0.005)	−0.288 (0.006)	−0.27 (0.004)
	IMD Q3	−0.381 (0.003)	−0.469 (0.007)	−0.575 (0.005)	−0.496 (0.005)	−0.514 (0.006)	−0.494 (0.005)
	IMD Q4	−0.472 (0.003)	−0.692 (0.007)	−0.764 (0.005)	−0.654 (0.006)	−0.699 (0.006)	−0.651 (0.005)
	IMD Q5 (least deprived)	−0.509 (0.004)	−0.789 (0.008)	−0.78 (0.005)	−0.758 (0.006)	−0.83 (0.007)	-0.681 (0.006)
		p < 0.0001	*p* < 0.0001	*p* < 0.0001	*p* < 0.0001	*p* < 0.0001	*p* < 0.0001
Slope (change in logged outlet density per year from baseline (2003))		−0.037 (0)	0.018 (0.001)	-0.05 (0)	0.069 (0)	0.145 (0)	0.032 (0)
		p < 0.0001	*p* < 0.0001	*p* < 0.0001	*p* < 0.0001	*p* < 0.0001	*p* < 0.0001
Effect on slope	IMD Q1 (most deprived)	ref.	ref.	ref.	ref.	ref.	ref.
	IMD Q2	0.012 (0)	0 (0.001)	0.007 (0)	−0.006 (0.001)	−0.013 (0.001)	−0.001 (0)
	IMD Q3	0.021 (0)	−0.004 (0.001)	0.014 (0)	−0.016 (0.001)	−0.036 (0.001)	−0.006 (0)
	IMD Q4	0.026 (0)	0.009 (0.001)	0.021 (0)	−0.02 (0.001)	−0.036 (0.001)	−0.008 (0)
	IMD Q5 (least deprived)	0.027 (0)	0.021 (0.001)	0.016 (0)	−0.021 (0.001)	−0.01 (0.001)	−0.014 (0)
		p < 0.0001	*p* < 0.0001	*p* < 0.0001	*p* < 0.0001	*p* < 0.0001	*p* < 0.0001
Region	Variance (intercept)	0.42	2.28	0.50	0.30	0.64	1.04
Postcode	Variance (intercept)	6.81	14.33	9.47	11.97	11.81	11.68
	Variance (slope)	0.01	0.04	0.02	0.02	0.03	0.02
	Covariance(intercept, slope)	−0.02	−0.18	−0.08	−0.03	−0.24	−0.14
Variance (residual)		0.24	1.32	0.55	0.85	0.99	0.55
